# Microbial composition of spiny ants (Hymenoptera: Formicidae: *Polyrhachis*) across their geographic range

**DOI:** 10.1186/s12862-017-0945-8

**Published:** 2017-04-05

**Authors:** Manuela Oliveira Ramalho, Odair Correa Bueno, Corrie Saux Moreau

**Affiliations:** 1 Universidade Estadual Paulista “Júlio de Mesquita Filho” UNESP – Campus Rio Claro, Biologia, CEIS. Av. 24A, 1515, Bela Vista, Rio Claro, SP 13506-900 Brazil; 2 Field Museum of Natural History, Department of Science and Education, Integrative Research Center, 1400 South Lake Shore Drive, Chicago, IL 60605 USA

**Keywords:** *Blochmannia*, *Wolbachia*, *Lactobacillus*, NGS, microbes, amplicon sequencing

## Abstract

**Background:**

Symbiotic relationships between insects and bacteria are found across almost all insect orders, including Hymenoptera. However there are still many remaining questions about these associations including what factors drive host-associated bacterial composition. To better understand the evolutionary significance of this association in nature, further studies addressing a diversity of hosts across locations and evolutionary history are necessary. Ants of the genus *Polyrhachis* (spiny ants) are distributed across the Old World and exhibit generalist diets and habits. Using Next Generation Sequencing (NGS) and bioinformatics tools, this study explores the microbial community of >80 species of *Polyrhachis* distributed across the Old World and compares the microbiota of samples and related hosts across different biogeographic locations and in the context of their phylogenetic history.

**Results:**

The predominant bacteria across samples were Enterobacteriaceae (*Blochmannia* - with likely many new strains), followed by *Wolbachia* (with multiple strains), *Lactobacillus,* Thiotrichaceae, *Acinetobacter, Nocardia, Sodalis,* and others*.* We recovered some exclusive strains of Enterobacteriaceae as specific to some subgenera of *Polyrhachis*, corroborating the idea of coevolution between host and bacteria for this bacterial group. Our correlation results (partial mantel and mantel tests) found that host phylogeny can influence the overall bacterial community, but that geographic location had no effect.

**Conclusions:**

Our work is revealing important aspects of the biology of hosts in structuring the diversity and abundance of these host-associated bacterial communities including the role of host phylogeny and shared evolutionary history.

**Electronic supplementary material:**

The online version of this article (doi:10.1186/s12862-017-0945-8) contains supplementary material, which is available to authorized users.

## Background

There are over 13,000 described species of ants belonging to the family Formicidae (Hymenoptera), which are widely distributed across the globe. The great diversity of the group is likely due to their ecological variability, including variation in nesting, feeding preferences and social behavior, and division of labor between castes [1, 2]. The genus *Polyrhachis*, Smith, 1857, is the fourth most species rich genus of ants and is characterized by its taxonomic, ecological and social diversity [3–5]. This genus contains more than 700 extant valid species [6], subdivided in the following recognized 13 subgenera: *Aulacomyrma* Emery, *Campomyrma* Wheeler, *Chariomyrma* Forel, *Cyrtomyrma* Forel, *Hagiomyrma* Wheeler, *Hedomyrma* Forel, *Hemioptica* Roger, *Hirtomyrma* Kohout, *Myrma* Billberg, *Myrmatopa* Forel, *Myrmhopla* Forel, *Myrmothrinax* Forel and *Polyrhachis* [5–7]. The genus *Polyrhachis* has a wide distribution across the tropical latitudes in the Old World, from Africa and Asia to Australia and a few Pacific islands, but being absent from Madagascar [7–9]. A possible reason for this restriction to the Old Word could be their late arrival to Africa, which potentially did not permit further dispersal to the New World as the continents had already drifted apart [10].

Nests of *Polyrhachis* can vary dramatically from terrestrial (present in the soil) to arboreal (in the canopy), in arid or tropical forests. Nests can be monodomous or polydomous, and colonies may be monogynous or polygynous (single or multiple breeding queens per nest). In addition colonies may vary in size from few to thousands of individuals [7, 11–13] with many species using larval silk to weave nests among plant leaves, a behavior that has been lost several times in the genus [4]. Additionally, *Polyrhachis* is one of the few examples from the subfamily Formicinae known to have semiclaustral colony foundation [14], where the queen will exit the nest during early colony foundation to forage in an attempt to obtain food resources, despite the danger of predation, unlike claustral nest foundation [15]. Recently Mezger and Moreau [10] in a large study (209 taxa) covering almost the entire distribution of the genus inferred the phylogeny and biogeography of the genus. Their molecular data support the monophyly of the genus, although some subgenera are not inferred as monophyletic. The authors were also able to estimate that the likely origin of the genus is South-East Asia, and that there were several dispersals into Australia, but only one to Africa.

In addition to the diversity of life history traits found across the ants, they also exhibit a range of associations with bacterial symbionts as seen in many other insect groups. For instance in an analysis across insect groups representing 63 species 76% were infected with associated bacteria [16]. In fact, Buchner [17] considered insects the model organismal group for the study of endosymbionts, since they coexist with microorganisms internally and externally to the body. Among the Hymenoptera, ants are well known for their associations with bacterial symbionts [18–20]. Diet flexibility exhibited by many species may explain much of the evolutionary success of the group, which is achieved in part due to the presence of endosymbionts that help improve host nutrition [21].

One well-studied example among the ants is the association of *Blochmannia* in the Camponotini ants, which circumscribes eight extant genera (*Calomyrmex*, *Camponotus*, *Echinopla*, *Forelophilus*, *Opisthopsis*, *Overbeckia*, *Phasmomyrmex*, and *Polyrhachis*) including *Polyrhachis,* the focal genus in this study. *Blochmannia* is a Proteobacteria specific to the Camponotini, which has been demonstrated to assist in providing essential amino acids to their host since their diets are defficient in nutrients as a consequence of their arboreal habitats [22, 23]. The nutritional role of *Blochmannia* is not the only beneficial aspect to the host, as it has been shown that *Blochmannia* also has the necessary genes to contribute to the metabolism of nitrogen, sulfur and lipids [24–26]. In addition to *Blochmannia* endosymbionts, among members of the Camponotini tribe, there are other species of endosymbionts that have been documented from these hosts, including *Arsenophonus* spp., *Cardinium hertigii*, *Hamiltonella defense*, and *Spiroplasma* spp. [27, 28]. However, little work has been done on the identification, diversity, and potential coevolution of bacteria associated with *Polyrhachis*, leaving many remaining questions about these associations including what factors drive host-associated bacterial composition.

To better understand the evolutionary significance of this association in nature, further studies addressing a diversity of hosts across locations are necessary. Therefore to address this question, we focus our study on the bacterial community of a host that exhibits high species diversity and a wide geographic distribution, to reveal more about the factors that influence bacterial communities. Leveraging next-generation sequencing, we document the diversity of bacteria associated with *Polyrhachis* (in 12 of the 13 subgenera), to identify the factors that structure the diversity of bacterial communities found across a diverse and widely distributed group of animals.

## Methods

### DNA extraction and bacterial DNA sequencing

For this study we included 142 samples of *Polyrhachis* representing 12 of the 13 subgenera from the study of Mezger and Moreau [10]. A complete list of samples used for this study can be found in Additional file 1: Table S1. The taxonomic identifications were determined by Mezger and Moreau [10] and vouchers were deposited in the collection of the Field Museum of Natural History, Chicago, USA during that study. Samples used for analyses were collected immediately into 95% ethanol in the field and and stored in 95% ethanol and kept at −20 °C until extraction of total DNA was performed. Total DNA was extracted from whole ant workers with Qiagen DNeasy Tissue kit following the manufacturer’s recommendations with slight modifications following Moreau [29] and we did not use the modification of the Quigen DNeasy kit for gram-positive bacteria. In addition, filtered pipette tips and sterile measurements were applied to avoid contamination of the samples, following recommendations of Moreau [29]. Amplicon sequencing of the microbial community was completed using the V4 region of 16S rRNA using primers described in Caporaso et al. [30], following the Earth Microbiome Project (EMP) protocol (515f primer and 806r; http://www.earthmicrobiome.org/emp-standard-protocols/16s/). PCR was performed in triplicate, each 25 μl PCR reaction contained 12 μl of MO BIO PCR Water (Certified DNA-free), 10 μl of 5 Prime HotMasterMix (1×), 1 μl of forward primer (5 mM concentration, 200 final pM), 1 μl Golay barcode tagged reverse primer (5 mM concentration, 200 pM final) and 1 μL of template DNA, under the following conditions 94 °C for 3 min to denature the DNA with 35 cycles at 94 °C for 45 s, 50 °C is 60 s, and 72 °C for 90 s, with a final extension of 10 min at 72 °C. After amplification, the triplicate reactions were combined (still maintaining the individuality of samples), and to confirm the efficiency of the reaction samples were visualized using gel electrophoresis (1%). The samples were quantified via qPCR and Qubit (Thermo Fisher Scientific) (see bacterial quantification section below), and only then pooled with different samples after controlling for volume (multiplex). For purification, only 100 μL of each pool was cleaned using the UltraClean PCR Clean-Up Kit (MO BIO), following the manufacturer’s recommendations. After quantification, the molarity of the pool is determined and diluted down to 2 nM, denatured, and then diluted to a final concentration of 6.1 pM with a 10% PhiX for sequencing on the Illumina MiSeq. A 151 bp × 12 bp × 151 bp MiSeq run was performed using the custom sequencing primers and procedures described in the supplementary methods in Caporaso et al. [30] on the Illumina MiSeq at the Field Museum of Natural History. All raw sequence data is available publicly in Figshare [https://figshare.com/s/290531bea3dee984444e] [31] and also available in the NCBI Sequence Read Archive (SRA) under accession number SRR5136256 and study SRP095836 [32].

### Bacterial quantification

To optimize Illumina sequencing efficiency, we measured the amount of bacterial DNA present with quantitative PCR (qPCR) of the bacterial 16S rRNA gene using 515f (5′ - GTGCCAGCMG CCGCGGTAA) and 806r (5′ - GGACTACHVGGGTWT CTAAT) universal bacterial primers of the EMP (http://www.earthmicrobiome.org/emp-standard-protocols/16s/). All samples and each standard dilution were analyzed in triplicate in qPCR reactions. All qPCRs were performed on a CFX Connect Real-Time System (Bio-Rad, Hercules, CA) using SsoAdvanced 2X SYBR green supermix (Bio-Rad) and 2 μL of DNA. Standard curves were created from serial dilutions of linearized plasmid containing inserts of the *E. coli* 16S rRNA gene and melt curves were used to confirm the absence of qPCR primer dimers. The resulting triplicate amounts were averaged before calculating the number of bacterial 16S rRNA gene copies per microliter of DNA solution (see Additional file 2: Table S5).

### Bioinformatic analysis

The sequences were analyzed in QIIME 1.9.1 [33]. First, the forward and reverse sequences were merged using SeqPrep. Demultiplexing was completed with the *split_libraries_fastq.py* command, commonly used for samples in fastq format. QIIME defaults were used for quality filtering of raw Illumina data. For calling the OTUs, we chose the *pick_open_reference_otus.py* command against the references of Silva 128 [34, 35] 97% identity with UCLUST to create the OTU table (biom format). Sequences with less similarity were discarded. Chimera checking was performed [36] and PyNAST (v1.2.2) was used for sequence alignment [37].

To test whether bacterial community composition is associated with taxonomic or geographic information, and if the taxonomic and geographic hierarchies can influence the bacterial community, we binned our data into different categories: “Subgenera” & “Species” to test taxonomic levels, and “Biogeography” & “Country”, to test the effect of geographic collection location. The *summarize_taxa_through_plots.py* command was used to create a folder containing taxonomy summary files (at different levels). Through this analysis it is possible to verify the total percentage of bacteria in each sample and subgenus. Additionally it is also possible to have a summary idea of the bacteria that constitute the bacterial community of *Polyrhachis*. In order to standardize sequencing effort all samples were rarefied to 400 reads. All samples that obtained fewer than 400 bacterial sequences were excluded from further analysis.

We used Analysis of Similarity (ANOSIM) to test whether two or more predefined groups of samples are significantly different, a redundancy analysis (RDA) to test the relationships between samples, and Adonis [38] to determine sample grouping. All these analyses were calculated using the compare_categories.py command in QIIME. The G test of independence (P, FDR_P and Bonferroni_P) was carried out to determine whether OTU presence/absence is associated with a host category through *group_significance.py* command. All these statistical tests serve to test whether the bacterial community is being influenced by any of the categories described above.

Alpha diversity was quantified using observed species richness, Shannon diversity, the Chao1 nonparametric richness estimator and whole-tree phylogenetic diversity and Simpson as implemented in equitability metric. We also compared alpha diversity based on a two-sample t-test using non-parametric (Monte Carlo) methods to test differences in OTU richness among subgenera. Unweighted and weighted UniFrac distance matrices [39], which uses phylogenetic information to calculate community similarity, were produced through the QIIME pipeline. The rarefaction curve was also created in QIIME and it is important to confirm if the sequencing was enough to cover the entire bacterial community associated with *Polyrhachis*. These beta diversity metrics were used to compare community level differences between categories. Jaccard dissimilarity metrics were calculated by beta_diversity.py command in QIIME. A matrix of community pairwise distances was generated by UniFrac and used to cluster samples by (i) the Unweighted Pair Group Method with Arithmetic Mean (UPGMA) method and (ii) principal coordinates analysis (PCoA). The UPGMA and PCoA analyzes that use the UniFrac beta diversity matrices show us which categories are influencing the bacterial community. As these analyzes have different methodologies and they will generate more robustness to the data of the study.

At a sequencing depth of 400, 64 samples passed this cutoff and were included in downstream analyses. To illustrate the relationship between ecological communities [40, 41], we implemented the analysis of multidimensional nonmetric scaling (NMDS) and related statistics in the PAST3 software package [42]. Sorensen (Dice coefficient) and Bray-Curtis similarity indices [40] were used to test the variation and the structure of the bacterial community, respectively. The samples were grouped according to the host subgenera, and after viewing the plots, analyzes of similarity (ANOSIM) with Bonferroni correction was used to determine statistical significance [40, 41, 43]. As this analysis requires at least two representatives from each group, the subgenera that had only one representative were grouped into a category “Mixed”.

Networks were visualized using Cytoscape3.2.1 [44] edge-weighted spring embedded algorithm to display the OTUs and sample nodes [45]. Each host-bacterial network was constructed as a graph, in which each node represented a host sample. Connections were drawn between samples representing the shared significant OTUs (each color represents a different OTU). Through the network it is possible to visualize the complexity that surrounds the bacterial community associated with *Polyrhachis* and to look for which category may best explain the pattern found. A heatmap was constructed with all OTUs that had 400 reads represented in the main dataset using heatmap.2 and the vegan package [46] in R [47]. The dendrogram of the samples shown in the heatmap was created with Bray-Curtis dissimilarity hierarchical clustering of bacterial communities in hclust. We also added a column dendrogram to cluster the genera that occur more often together. In this analysis we restrict only the most well represented OTUs and check if there is any OTU specificity within any of the categories described above. With this analysis it is also possible to verify the samples that have multiple infections as can happen with specimens infected with *Wolbachia* and *Blochmannia* [48].

We did analyses of correlation and coevolution: 1) compared the bacterial community following the host phylogeny of Mezger and Moreau [10] (coevolution/vertical transfer); 2) and similarity of bacterial community from hosts based on their locality (horizontal transfer). For this, geographic distances were calculated from sample locality information using geographical collection coordinates (latitude/longitude) of each included sample. They were transformed to UTM distance metric using the “rgdal” package [49] in R [47] and geographic distance matrix was constructed. The weighted distance of all sample were calculated through beta diversity in QIIME. The correlation between the bacterial community and geographic distances of *Polyrhachis,* and bacterial community and host phylogeny were calculated using the Mantel test (999 permutations) using the “vegan” package [46] in R. We also tested for significant associations between bacterial community dissimilarities and host genetic and geographic distances, we used partial Mantel tests, as implemented in the vegan package in R [46].

## Results

### Bacterial 16S rRNA diversity

Illumina 16S rRNA sequencing of *Polyrhachis* ant hosts reveals a relatively simple microbiota that is remarkably conserved. Our analyses obtained 5443 observed OTUs from a total of 61,225 reads from 132 specimens from 12 of the 13 subgenera of *Polyrhachis* collected from across the Old World, which permitted analyses comparing different host categories: species, subgenera, biogeography and countries.

**Fig. 1:**
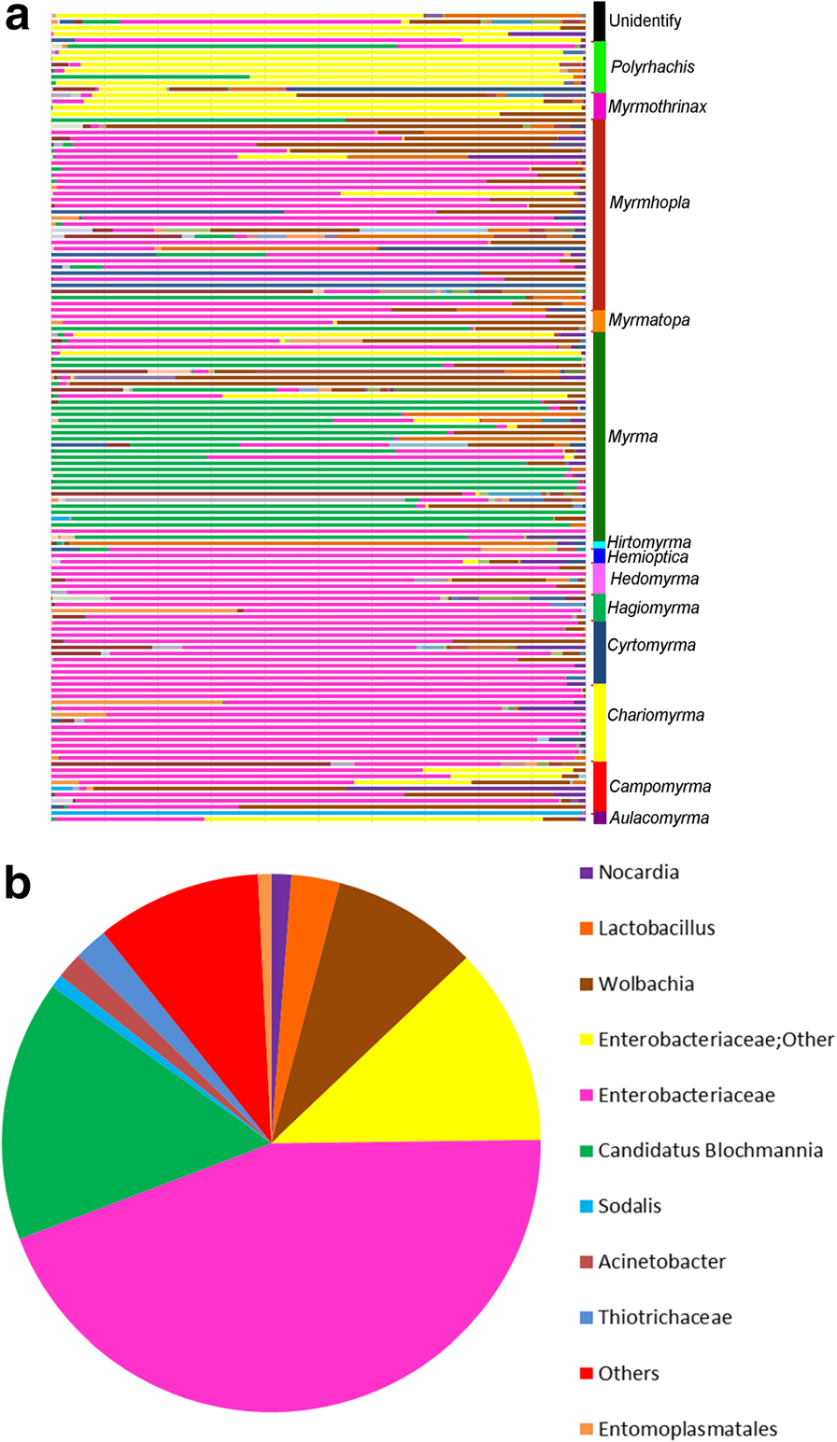
Summary table of bacterial OTUs found in *Polyrhachis* samples with 16S rRNA amplicon sequencing. **a** *Polyrhachis* subgenera used in this study and their bacterial communities. Bar graphs for each library (one column = community from a single worker) show the percentage of sequence reads classified to selected 97% OTUs. Each color represents a distinct bacterium. The samples were grouped according to the subgenera which they belong. **b** Summary of all OTUs found in this study with legend ordered in proportion of reads found across all 132 samples. The relative abundance of reads at the taxonomic level of bacteria is displayed. Orders that accounted for less than 0.8% in a sample are summarized in a category termed “Other”

The diversity and the total number of bacteria found in *Polyrhachis* are represented in Fig. 1. Our analyses recovered variation from 1 to a maximum of 1384 OTUs of bacteria per sample, a lower absolute diversity compared to other herbivorous ants such as *Cephalotes* [20, 50–52]. The predominant bacteria across samples were Enterobacteriaceae (44.40%), *Candidatus Blochmannia* (15.70%), Enterobacteriaceae - other (11.90%), *Wolbachia* (8.80% - multiple strains) and *Lactobacillus* (2.90%), followed by Thiotrichaceae (2.0%), *Acinetobacter* (1.60%), *Nocardia* (1.20%), *Sodalis* (0.80%) and Entomoplasmatales (0.80%) [Additional file 3: Table S2].

### Statistical analyses of bacterial community diversity

We performed statistical tests (weighted and unweighted) to examine potential patterns that influence the bacterial community of *Polyrhachis*. From these we found subgeneric taxonomic affliation of the host (Adonis, unweight R^2^ = 0.23602 and *P* = 0.002; Anosim, unweight R^2^ = 0.11400 and *P* = 0.029; RDA, unweight Pseudo F = 1.47656 and significance = 0.001) had more influence on bacterial community composition than broader biogeographic origin, country or species, although not statistically significant.

Through the results of the G test (P, FDR_P and Bonferroni_P), we found bacteria community presence/absence is significantly different across multiple categories (species, subgenera, biogeography and country) [see in Additional file 4: Table S3]. Within the species category more bacteria were significant across samples than the other host categories. However, the bacteria Enterobacteriaceae (multiple strains, including *Candidatus Blochmannia*), *Wolbachia* (multiple strains), *Nocardia*, *Sodalis*, Thiotrichaceae and *Lactobacillus* were significant across all categories [Additional file 4: Table S3].

### Alpha diversity

**Fig. 2:**
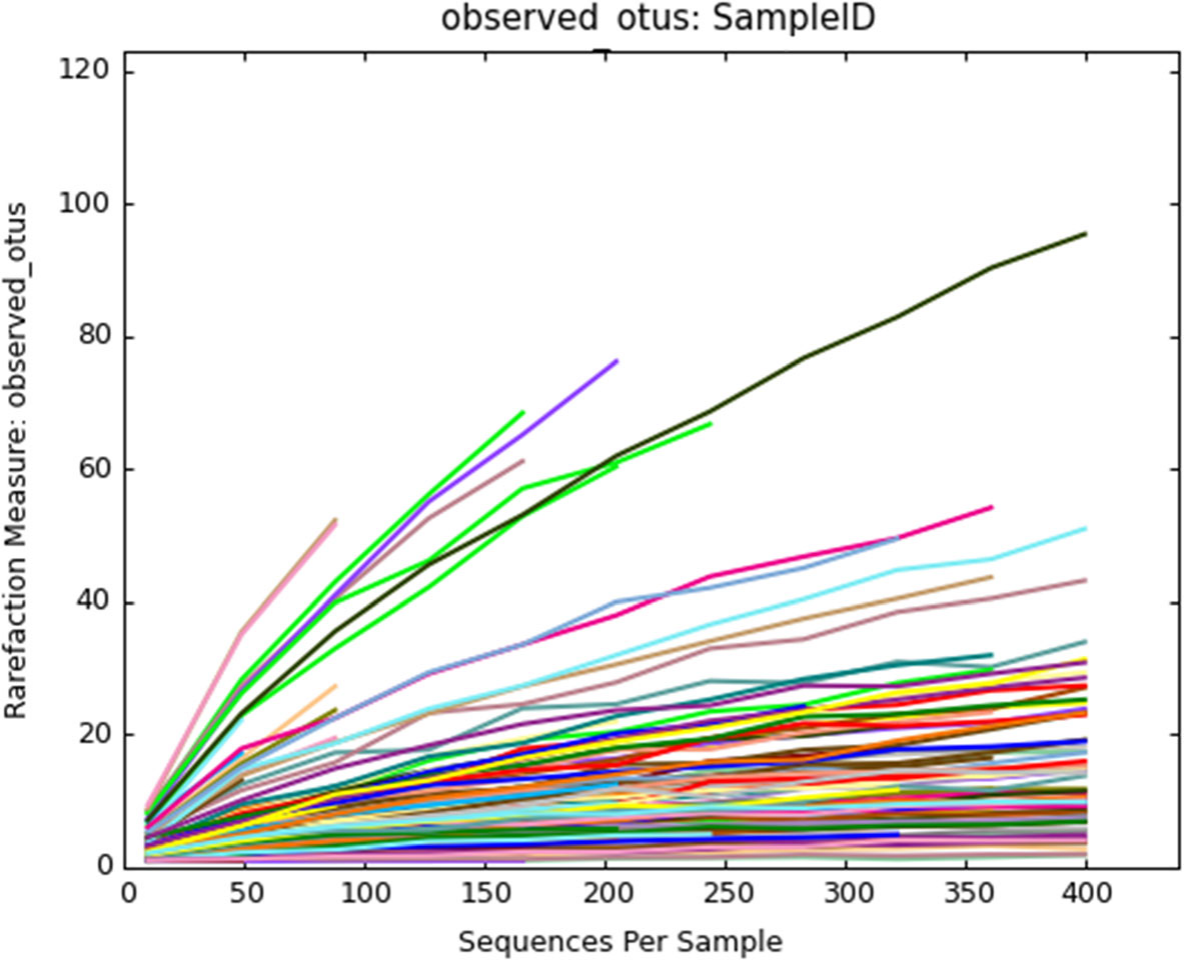
Rarefaction curves were used to estimate richness in the observed OTUs. The vertical axis shows the bacterial OTUs observed and the number of sequences per sample is shown on the horizontal axis. Note that although sequencing covers thousands of Illumina reads, some samples have not reached the plateau

Alpha diversity (Chao1, PD whole tree, observed OTUs, Simpson and Shannon) observed across *Polyrhachis* individuals was not high. For the remaining samples at sequencing depth of 400, we recovered high variation of diversity [Additional file 5: Table S4]. Likely due to the small amount of sequence for these samples, we did not obtain significant results when comparing differences in OTU richness among host subgenera. Through the rarefaction curve analysis of observed OTUs, our sequencing coverage of the bacterial communities appears satisfactorily for most samples, but even with the thousands of Illumina sequence reads, sampling was not sufficient to achieve a plateau for all specimens (Fig. 2).

### Beta diversity

**Fig. 3:**
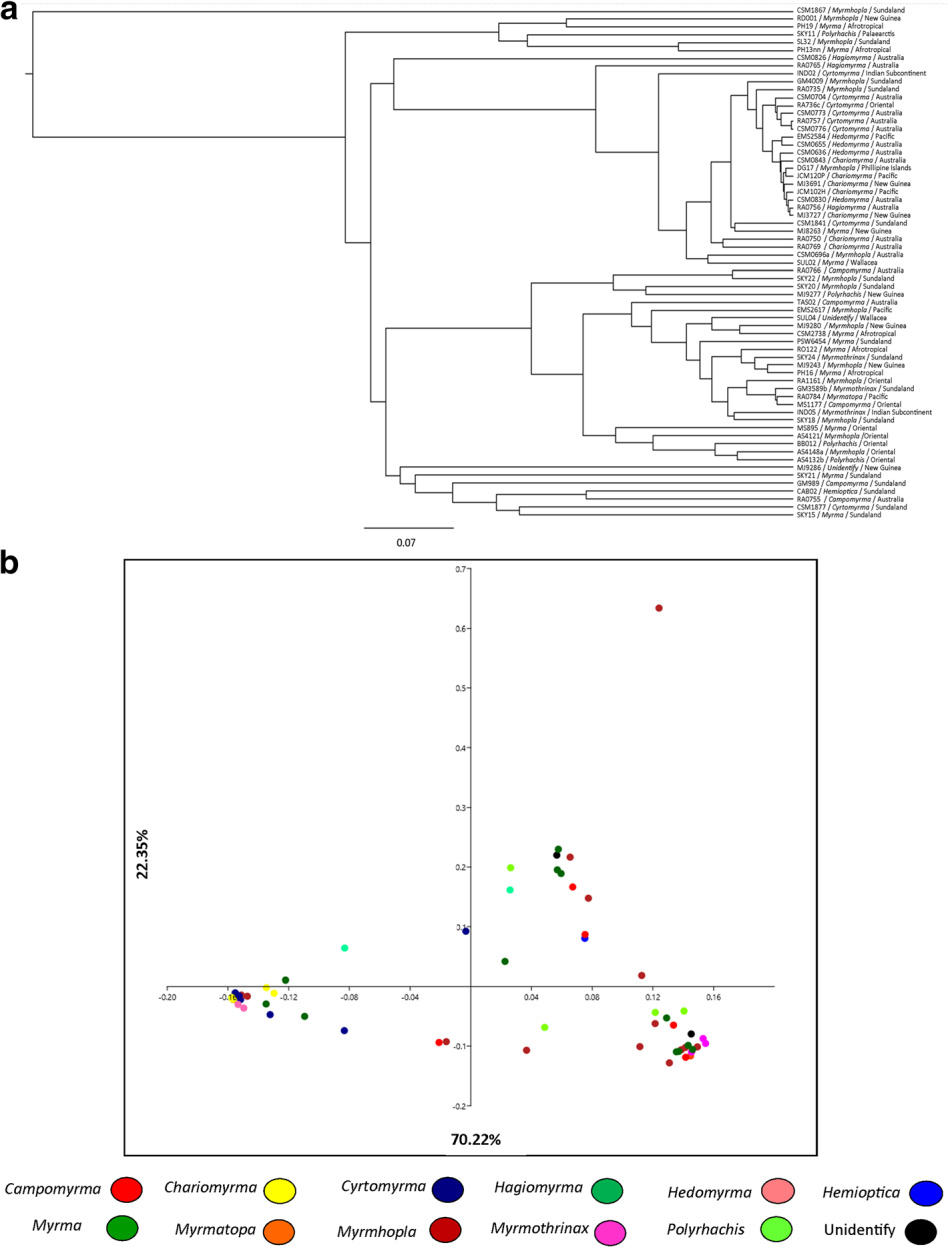
Beta diversity found in *Polyrhachis* samples rarefied to a read depth of 400 (50% of samples). Note that after this depth only 64 samples remained. **a** UPGMA tree (unweighted UniFrac method) of the entire bacterial community of *Polyrhachis*. Through the tree it is possible to visualize that were grouped samples of several subgenera and distinct localities. **b** PCoA plots (weighted UniFrac method) of bacterial communities associated with *Polyrhachis* at the 97% OTU level. Axis 1 = 70.22% and axis 2 = 22.35%. The dots were colored according to the subgenera they belong to. Note that although not fully clustered, there is a certain ordering of subgenera, indicating that host phylogeny plays an important role in structuring the bacterial community

**Fig. 4:**
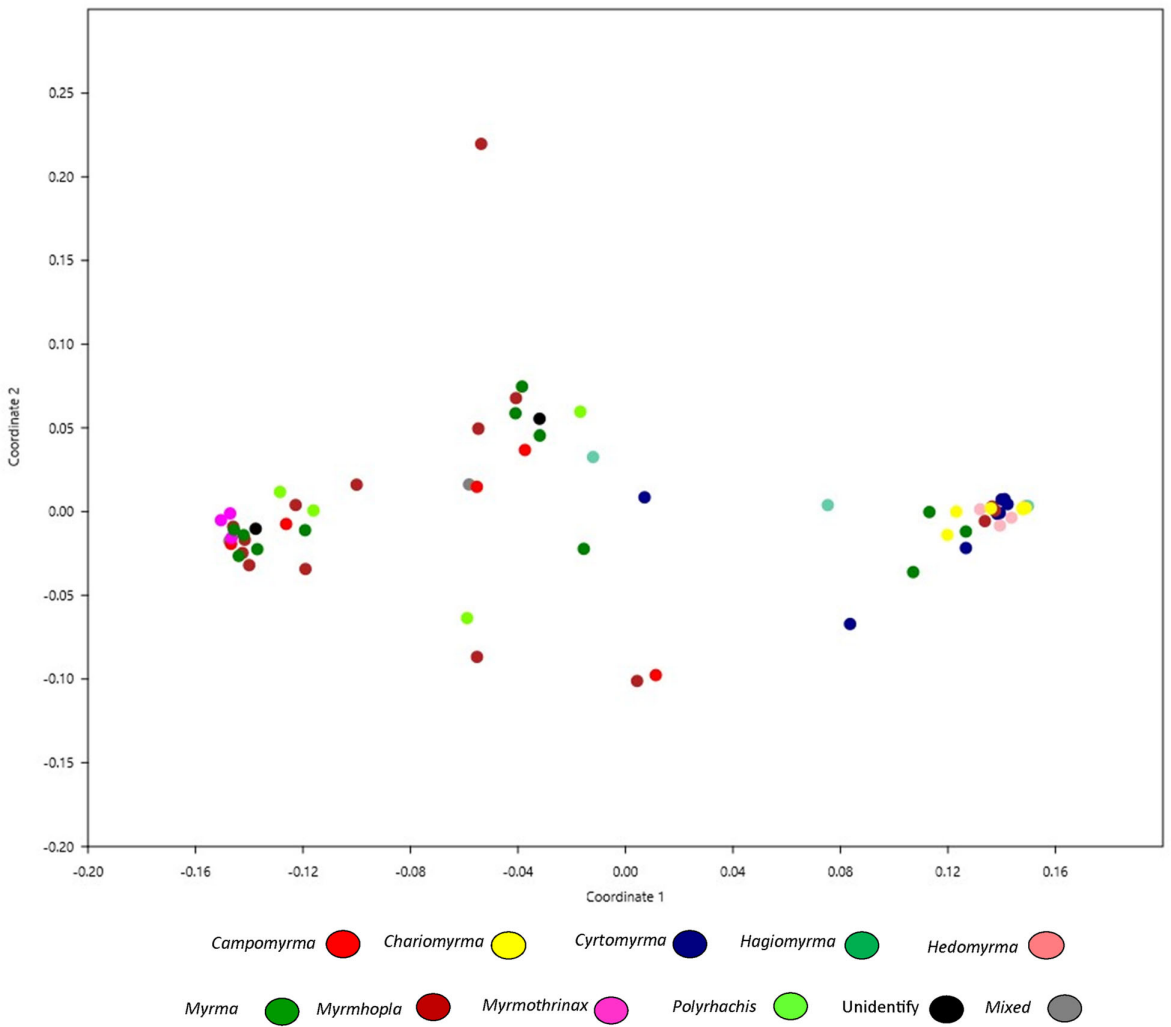
Nonmetric multidimensional scaling (NMS) plot illustrating bacterial community structure among *Polyrhachis* subgenera (Bray-Curtis similarity index). As this analysis requires at least two representatives from each group, the subgenera that had only one representative were grouped into a category “Mixed”. The distance between bacterial communities represents their underlying distance in the multivariate space. Axis 1 = 0.901, axis 2 = 0.03547, and stress 0.044, a good indicating value, which means that the analysis of NMs obtained an informative representation of the bacterial community. The dots were colored according to the subgenera they belong. Note that although complex, there is a structure of subgenera

Through analysis of beta diversity (matrices UniFrac weighted distance, depth 400 (50% of samples)) we find similarity of the bacterial communities from these samples. The UPGMA tree (Weighted UniFrac method) of the entire bacterial community of *Polyrhachis* grouped samples of different subgenera and biogeography, but we realized that the samples were grouped according to high infection of different bacteria (Figs. 3a and 4). Variation among samples in their bacterial taxonomic composition was visualized using constrained principal coordinates analyses (Fig. 3b). The average Jaccard dissimilarity metric was 0.91, which suggests only a few bacterial community members were shared among all individuals of *Polyrhachis*. Also, we found no significant changes in the composition (Soresen index) of the bacterial community of *Polyrhachis* (*R* = 0 and *P* = 1). That is, different subgenera do not have significantly different bacteria. But there was an effect of the structure of the bacterial community (Bray-Curtis index, stress 0.044, *R* = 0.2205 and *P* = 0.0003) when all subgenera were compared. In the analysis of the subgenera in pairs, it was not possible to identify significant results.

### Network analysis

**Fig. 5:**
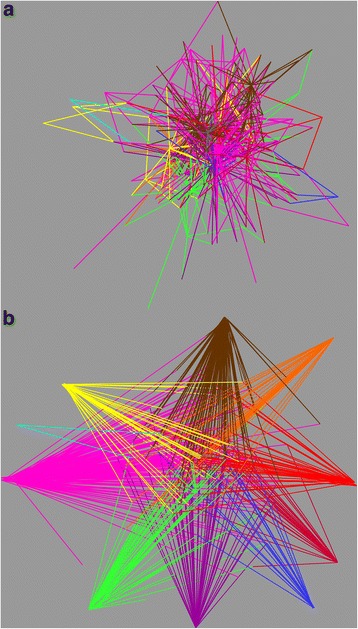
Network analysis of Polyrhachis with edges representing the main community bacterial members. The edges were colored according to the different shared bacteria: Nocardia (purple), *Lactobacillus* (orange), *Wolbachia* (brown), Enterobacteriaceae (pink), Enterobacteriacea - other (yellow), *Candidatus Blochmannia* (green), *Sodalis* (light blue), Thiotrichaceae (dark blue), and Others (red) **a** Default parameters of Spring-embedded edge-weighted algorithm. The host nodes are not visible for easy viewing, but can still be represented by each vertice. In this analyze the vertices (host) that share more OTUs appear close together. Note how complex the bacterial community network is. **b** Default parameters manually edited. After this edition, it is possible to visualize which bacteria are better represented in this network. As in this case, the bacterium Enterobacteriaceae in pink is highly represented in the bacterial community of *Polyrhachis*

To examine the connection between samples with shared significant OTUs, we used Cytoscape to construct a network graph in which each node represented a host sample. Network analyzes were performed using default parameters using the spring-embedded edge-weighted algorithm (Fig. 5a), and the spring-embedded edge-weighted algorithm manually edited (Fig. 5b), which approaches the samples according to the number of OTUs shared. OTUs with less than 400 reads were hidden for easy viewing. In this analysis, only the edges of Enterobacteriaceae (pink), Enterobacteriacea, other (yellow), *Candidatus Blochmannia* (green), *Wolbachia* (brown), *Lactobacillus* (orange), *Nocardia* (purple), *Sodalis* (light blue), and Thiotrichaceae (dark blue), Others (red) were colored. Note how complex these associations are (Fig. 5).

### HeatMap

Through heatmap analysis (bacterial genera and family levels), we investigated the entire bacterial community found in this study and the abundance of OTUs found in each sample. For easy viewing, we choose to show only OTUs with more than 400 reads. It is interesting to note that more than 50% of the bacterial community consisted of Enterobacteriaceae (multiple strains). Several strains of Enterobacteriaceae were restricted to specific subgenera of *Polyrhachis*. This includes *Candidatus Blochmannia*-New.ReferenceOTU70 which was almost exclusively associated with the host subgenus *Myrma* from the Afrotropics, Enterobacteriaceae-New.ReferenceOTU13 which was almost exclusively with subgenus *Polyrhachis,* and Enterobacteriaceae-New.CleanUp.ReferenceOTU0 is found in samples from subgenus *Myrmhopla.*

**Fig. 6:**
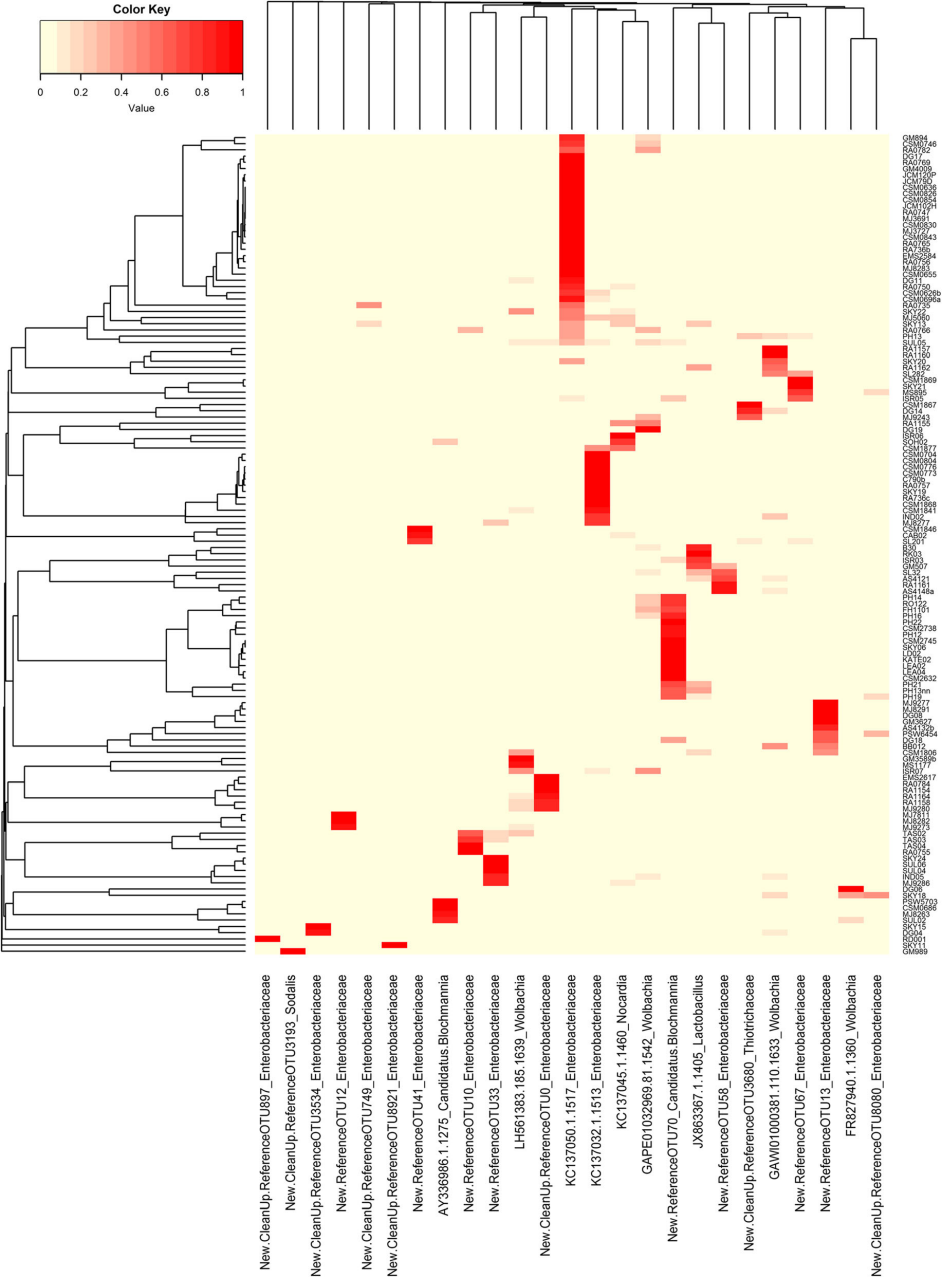
The colors in the heatmap indicate variation in the relative abundance of different bacteria in *Polyrhachis*, ranging from 0% (light yellow) to 100% (red). Dendrograms were generated from Bray–Curtis distance matrices. For easy viewing, we choose to show only OTUs with more than 400 reads Note there are strains of Enterobacteriaceae restricted to specific subgenera of *Polyrhachis*, such as *Candidatus Blochmannia*-New.ReferenceOTU70 with *Myrma* from the Afrotropics, Enterobacteriaceae-New.ReferenceOTU13 with *Polyrhachis,* and Enterobacteriaceae-New.CleanUp.ReferenceOTU0 with *Myrmhopla.* In this analysis the presence of multiple *Wolbachia* infections in some Polyrhachis samples is also evident

Another interesting observation is there are four different highly abundant *Wolbachia* strains found across our samples. We observed an infection rate of 49.24% from across our 132 samples. There are even multiple individuals (*n* = 25, 38.46%) with the presence of a double infection of *Wolbachia*. Also, the presence of *Lactobacillus* was unexpected and was identified from samples from across the distribution of the genus (Fig. 6).

### Correlation and coevolution tests

The Mantel test verified the correlation of the bacterial community and geographic distance when analyzed with phylogenetics information from Merzer and Moreau [10] for *Polyrhachis* hosts. In addition using the Mantel test we found support for correlation between the phylogeny of the host and the bacterial community using the “vegan” package [46]) in R (*R* = 0.2289 and *P* = 0.0001). We also tested for the influence of locality on the bacterial community sampling, again using the Mantel test through the R software package to generate the pairwise geographical distances of each *Polyrhachis* sample. Our results showed that there is no correlation between the geographical location and the bacterial community overall (*R* = 0.08582 and *P* = 0.0756). Lastly through a partial mantel test of the three matrices (bacterial community, host phylogeny and geographical distances), we were able to demonstrate that the phylogeny of the host explains just part (*R* = 0.2279 and *P* = 0.0001) of the entire bacterial community, while geographical distance does not have significant influence on structuring the bacterial community of spiny ants (*R* = 0.09075 and *P* = 0.0697). While conducting more specific analysis of correlations of individual OTUs with the phylogeny of the host, we did not obtained significant results.

## Discussion

The use of NGS technologies to study the microbiome is relatively recent and these data are providing an unprecedented understanding of microbial diversity and putative function in many habitats and across a diversity of hosts. The bacterial communities associated with hosts can vary from simple to complex and can be influenced by environmental, genetic and other factors of the host or host’s environment which can make the task of understanding the elements determining host-association a challenge [53]. The mechanisms that govern the ecology and evolution of the microbiota inside most hosts are still unknown and detailed studies are limited [27, 45, 51–56]. Besides revealing the bacterial community associated with hosts, studies that attempt to explain changes and what factors influence this bacterial community are still scarce [57]. Many factors may influence the microbiota associated with the host, for example: diet, pH, host phylogeny (coevolution), life stage, and host location [58–62]. Of all these factors, the phylogeny of the host and diet has a strong effect on bacterial communities for many hosts [45]. In a study involving ants, Anderson et al. [63] found similarity of the bacterial communities between species of the same trophic level, and found differences between herbivorous and predatory species. However, geographic location can also be an important mechanism influencing the microbiome [53]. Our results are the first to characterize the bacterial community associated with the diverse spiny ant genus *Polyrhachis* from across their distributional range. Additionally, we were able to test whether the host phylogeny or biogeography could be influencing the diversity of bacterial communities found associated with this animal group.

Our results highlight how complex associations of different bacteria associated with *Polyrhachis* can be. This suggests that the evolutionary history of the host can influence the bacterial community in *Polyrhachis*. Ley et al. [45] who analyzed mammalian bacterial communities found correlations between diet and host microbiota, which they related to the gut physiology of the host. Compared to vertebrates, insects have a lower diversity of gut bacterial communities and these can be more variable [64, 65], which makes the understanding of the mechanisms that may influence communities difficult.

In a study analyzing various insects Jones et al. [55] also recovered low bacterial richness, as has been found in other studies [66–68]. One possible explanation is that the host has a mechanisms to prevent the establishment of new bacteria, as a way to defend against pathogens [64]. Although the high infection with *Wolbachia* found in this study could also be an explanation for the low richness found in *Polyrhachis*, since this bacteria can reduce the diversity of bacterial communities [69].

### Composition of the bacterial community

The bacteria most commonly found in our study were Enterobacteriacea (multiple strains). It was found present in all sampled individuals (at least one strain) across different subgenera of *Polyrhachis* ants, sampled from across their known geographical range (Fig. 1). *Blochmannia*, a member of the Enterobacteriacea, is known to possess primary interactions in Camponotini ants, which includes *Polyrhachis*. For symbionts of *Polyrhachis* the phylogenetic trees are congruent with those of their hosts across long periods of evolutionary time, indicating the coevolution of host and symbiont in previous studies [70–72] and the current study. In fact in previous studies this endosymbiont was recovered as a monophyletic group associated with Camponotini ants, showing coevolution of host and endosymbiont and suggests the acquisition of this microorganism must have occurred in the common ancestor of this ant tribe [22, 23].

The *Blochmannia* endosymbiont is known to play a nutritional role for the host, providing several essential amino acids [73], especially in early life [24, 25, 74]. *Blochmannia* also maintains certain genes for basic cellular functions, such as biosynthesis of the nine essential amino acids (excluding Arginine), and urease cofactors and enzymes, which allows the symbiont to recycle urea nitrogen provided by the host’s excretory system [73, 75, 76]. In addition, the nutritional role of *Blochmannia* is not the only potential interaction with its host, as it has also maintained genes needed to contribute to the metabolism of nitrogen, sulfur and lipids [24–26].

Overall we detected low *Candidatus Blochmannia* abundance, contrary to what we expected based on previous studies from this ant genus [22, 23]. But *Blochmannia* are known to have high mutational rates [77], suggesting that many if not most of the bacteria only identified as “Enterobacteriaceae” or “Enterobacteriaceae - other” may in fact be *Blochmannia*. This high mutation rate and the relatively short fragment of 16S rRNA that can be sequenced using NGS methods is likely responsible for our inability to assign most Enterobacteriaceae to lower taxonomic categories.

When we restricted our analysis to the bacterial genus level, 15.70% of samples included *Candidatus Blochmannia*. When we reduced the hierarchical level to Family, we recovered Enterobacteriaceae in more than 70% of all bacterial communities across geographical localities and host subgenera, with all individuals having at least one OTUs from this family. We also found some strains of Entecobacteriacea associated with specific host subgenera. This is potentially indicative of co-evolution and specificity of the strain to the host. For example we found *Candidatus Blochmannia*-New.ReferenceOTU70 associated with subgenus *Myrma* from the Afrotropics, Enterobacteriaceae-New.ReferenceOTU13 associated with *Polyrhachis*, and Enterobacteriaceae-New.CleanUp.ReferenceOTU0 associated with *Myrmhopla*.

This may suggest *Blochmannia* has undergone rapid change since its mutational rate is known to be high [77], which could prevent the identification of these OTUs as *Blochmannia*. Previous studies from the tribe Camponotini using traditional molecular techniques, i.e. Sanger sequencing of the entire 16S rRNA, showed a strong relationship of this bacterium with the host tribe [22, 23]. Even assuming that all Enterobacteriaceae found in this study belong to the bacterial genus *Blochmannia*, our data is still without precedent, since Brown and Wernegreen [78] using NGS in a study involving *Camponotus* found that *Blochmannia* typically constituted 95–98% of reads, and in our study of *Polyrhachis* only 70% (*Blochmannia* and all OTUs of Enterobacteriaceae combine). This lack of sequence conservation suggests that this bacterium may not be preforming these same fundamental roles suggested by previous studies, at least for the genus *Polyrhachis*. More studies are needed to reveal the function of these bacteria in the genus.

Although our results suggest that even without the modification of the Qiagen DNeasy kit for gram-positive bacteria, our DNA extraction method was able to obtain some DNA from gram-positive bacteria, but this could still influence the diversity of bacteria we are able to detect and our method may be omitting some gram positive bacteria. One interesting finding we uncovered is regarding selection of reference options for calling OTUs in Silva 128 [34, 35]. Initially we chose the pick_closed_reference_otus.py command instead of pick_open_reference_otus.py command, but this greatly reduced the number of bacteria sampled in our study. Through this command the aligned sequences are compared to the reference database, and if it does not match with any reference, the sequence was excluded from the analysis. In other words, the use of this command is not able to identify novel diversity, being restricted to already-known taxa [79]. As it is known that *Polyrhachis* have *Blochmannia* [22, 23], and this bacterium has a high mutational rate [77], the pick_open_reference_otus.py command enabled the detection of unknown OTUs (i.e., those that are not represented in the reference database) compared with the closed reference of Silva 128 [34, 35]. The open reference option was able to find 429 additional OTUs (New.Reference and New.CleanUp.Reference). And when we limited our search to only OTUs with over 400 reads of 25 OTUs that met these criteria, 16 were new (Fig. 6). With that in mind, we strongly suggest that in cases where high bacterial mutational rate is known, the use of open reference instead of closed reference to insure detection of bacterial diversity is advised.

Other studies have shown *Wolbachia* as a major player within the bacterial community of invertebrates [19, 27, 28, 55, 78]. For example, in the screening of 24 *Polyrhachis* species, five (20.8%) were infected with *Wolbachia* [27]. Kautz et al. [28] found *Wolbachia* in 25% of *Polyrhachis* analyzed from Australia. In our analysis we found *Wolbachia* in 65 samples of *Polyrhachis* (49.24%), and of these samples 25 showed multiples strain infections (38.46%). All strains have a wide distribution across our samples of *Polyrhachis*. Although *Wolbachia* is known for manipulating the reproduction of the host, its function in ants is still unclear.

The next most common bacteria associated with *Polyrhachis* is *Lactobacillus* found in 31 samples (23.48%). This bacterium was found widely distributed across host subgenera and across host locations. Recent work in the ants has shown the presence of *Lactobacillus*, but its function in this group is not yet fully understood. Kellner et al. [80], also through NGS techniques, found 56% of their samples of *Mycocepurus smithii* (a fungus-farming ant) contained Lactobacillales. *Lactobacillus* have antimicrobial properties and are widely used in the food industry and fermentation of milk products [81]. *Lactobacillus* expresses antimicrobial properties through lactic acid secretion to acidify environmental conditions that some other bacteria and fungi cannot tolerate. Therefore Kellner et al. [80] believe that *Lactobacillus* may serve an important role as defense pathogens in the *M. smithii* system. In another study involving termites, *Lactobacillus* was found in the insect feces where, in addition to this protection function, it can also serve as a substrate or fertilizer [82, 83].

*Polyrhachis*, along with a few other ant genera, is known for the absence of a metapleural gland [84]. Four possible functions are assigned to this gland: antimicrobials, chemical defense, recognition odor and territorial marking. The first two functions are well accepted and supported by several studies, while the last two require further investigation [85]. This gland is essential for ground nesting ants, since they are more susceptible to infections due to the dark and sometimes damp conditions of their nesting habitat. Although many species of *Camponotus* and *Polyrhachis* nest arboreally, those with terrestrial habits should have evolved alternative antimicrobial defenses [86]. Based on this hypothesis, another study suggested that the behavior of self-cleaning, as well as the use of venom with antimicrobial properties, are the key to disease resistance within the colony of a weaver ant species of *Polyrhachis dives,* Smith [87]. With this in mind, *Lactobacillus* could be assisting in the defense of the colony potentially replacing the role of the metapleural gland for this genus.

In our findings Entomoplasmatales is present in only 0.80% of the bacterial community found in *Polyrhachis*. This result is different than those previously reported in the literature, as Kautz et al. [28] observed 46% infection rate by *Spiroplasm* (Entomoplasmatales) and Russell et al. [27] found 20% infection by *Spiroplasm* for this genus. Russell et al. [27] also suggested that *Spiroplasm* enrichment could be a feature specific to *Polyrhachis* and their close relatives. This may not be a genus-wide attribute, because four of the six *Polyrhachis* included in their study were from the Australian Wet Tropics and came from species in the subgenus *Chariomyrma* (4/6 species infected). Our findings do not support this as we did not find *Spiroplasm* strongly associated with *Polyrhachis*, even within the subgenus *Chariomyrma*.

The correlation (partial mantel and mantel tests) found in this study indicates that host phylogeny (vertical transfer) could influence the bacterial community to some extent. Our statistical tests also gave similar results to those observed for the mantel tests, suggesting that the phylogeny of the host (subgenera) explains part of the bacterial community, and host location (country or biogeography) none. This result corroborates Meirelles et al. [88] that also did not find any geographic signature in the bacterial community from the fungus-growing ant, *Atta texana* (Buckley). Certainly the specificity found in some strains of Enterobacteriaceae within subgenera of *Polyrhachis* contributed to our findings of correlation between bacterial community and phylogeny of the host (vertical transfer). All these data provide support for the coevolution of *Polyrhachis* and their microbiome, since geography can be seen as an approximation to the sum of environmental effects, such as local weather patterns and availability of food sources, which select for and influence local community assemblages. But we cannot assume that horizontal transfer does not also contribute to the diversity of bacterial communities found. Our findings of what drives the bacterial community of *Polyrhachis* corroborates the findings of Sanders et al. [52] and Ley et al. [45]. The microbiota found in these studies also demonstrated that there is a significant effect of phylogeny of the host. Therefore, although there is a difference (both in abundance and diversity) between bacterial communities of different ants we still understand very little about the mechanisms that influence the microbiome.

## Conclusions

These results of varing infection rates of *Polyrhachis* by a diversity of bacteria demonstrate the power of next-generation sequencing to uncover host-associated bacteria. In addition, our data uncovered novel bacteria, showing that with this technique it is possible to explore and discover bacterial diversity never before studied from hosts. We also recovered some species or groups of bacteria associated with only one host subgenus suggesting host-specificity and host-phylogeny could be a determining factor in the distribution of bacterial community in these associations. Furthermore, we did not recover any patterns of bacterial diversity correlated with a specific host geographic region, suggesting these microbes are not just being picked up in the environment. In the general context, we observed the complexity of an entire bacterial community associated with *Polyrhachis* throughout their geographic range. We focused our discussion on the most commonly recovered bacteria because we believe that these bacteria described above have an important role and may be able to influence the evolution and ecology of the host. General knowledge about the host united with information on the host’s microbiome are important tools to understand more about the evolutionary complexity of these associations in nature.

## Additional files

Additional file 1: Table S1. Specimens of *Polyrhachis* used in this study. (XLSX 18 kb)

Additional file 2: Table S5. Bacterial Quantification through 16S rRNA gene (qPCR) of all Polyrhachis samples. Each sample was analyzed in triplicate therefore follows the values of average and standard deviation for each sample. (XLSX 15 kb)

Additional file 3: Table S2. Percentage of the most common bacteria found in *Polyrhachis* samples. (XLSX 11 kb)

Additional file 4: Table S3. Analysis of G test. G test of independence (P, FDR_P and Bonferroni_P) across *Polyrhachis* samples to determine whether OTU presence/absence is associated with different host categories. (XLSX 18 kb)

Additional file 5: Table S4. Alpha diversity estimation. Chao1, PD whole tree, Observed OTUs, Simpson and Shannon observed in *Polyrhachis* individuals. (XLSX 21 kb)

## Abbreviations

EMP: Earth microbiome project
NGS: Next generation sequencing
NMDS: Multidimensional nonmetric scaling
OTU: Operational taxonomic unit
PD: Phylogenetic diversity
RDA: Redundancy analysis
UTM: Universal transverse mercator

## References

[1] Wilson EO (1987). Causes of Ecological Success: The case of the ants. J Anim Ecol.

[2] Hölldobler B, Wilson E (1990). The ants.

[3] Van Zweden JS, Carew ME, Henshaw MT, Robson SKA, Crozier RH (2007). Social and genetic structure of a supercolonial weaver ant, *Polyrhachis robsoni*, with dimorphic queens. Insect Soc.

[4] Robson SKA, Kohout RJ, Beckenbach AT, Moreau CS (2015). Evolutionary transitions of complex labile traits: Silk weaving and arboreal nesting in *Polyrhachis* ants. Behav Ecol Sociobiol.

[5] Kohout RJ (2010). A review of the Australian *Polyrhachis* ants of the subgenera *Myrmhopl*a Forel and *Hirtomyrma* subgen. Nov. (Hymenoptera: Formicidae: Formicinae). Mem Queensl Museum Nat.

[6] Bolton B. An online catalog of the ants of the world. 2016 [cited 2016 Oct 20]. Available from: http://www.antcat.org/

[7] Dorow WH (1995). Revision of the ant genus *Polyrhachis* Smith, 1857 (Hymenoptera: Formicidae: Formicinae) on subgenus level with keys, checklist of species and bibliography.

[8] Fisher BL (1997). Biogeography and ecology of the ant fauna of Madagascar (Hymenoptera: Formicidae). J Nat Hist.

[9] Guenard B, Weiser M, Economo E (2014). Ant global diversity: opening new possibilities in ant-biology.

[10] Mezger D, Moreau CS. Out of South-East Asia: Phylogeny and biogeography of the spiny ant genus *Polyrhachis* Smith (Hymenoptera: Formicidae). Syst Entomol. 2016;41:369–78.

[11] Robson SKA, Kohout RJ (2005). Evolution of nest-weaving behaviour in arboreal nesting ants of the genus *Polyrhachis* Fr. Smith (Hymenoptera: Formicidae). Aust J Entomol.

[12] Nielsen MG (1997). Nesting biology of the mangrove mud-nesting ant *Polyrhachis sokolova* Forel (Hymenoptera, Formicidae) in northern Australia. Insect Soc.

[13] Dorow WH, Kohout RJ (1995). A review of the subgenus *Hemioptica* Roger of the genus *Polyrhachis* Fr. Smith with description of a new species (Hymenoptera: Formicidae: Formicinae). Zool Med.

[14] Lenoir A, Dejean A (1994). Semi-claustral colony foundation in the formicine ants of the genus *Polyrhachis* (Hymenoptera: Formicidae). Insect Soc.

[15] Nickele MA, Pie MR (2013). Reis Filho W, Penteado S do RC. Formigas cultivadoras de fungos: estado da arte e direcionamento para pesquisas futuras. Pesqui Florest Bras.

[16] Jeyaprakash A, Hoy MA (2000). Long PCR improves *Wolbachia* DNA amplification: wsp sequences found in 76% of sixty-three arthropod species. Insect Mol Biol.

[17] Buchner P (1965). Endosymbiosis of animals with plant microorganisms. Endosymbiosis Anim. with Plant Microorg.

[18] Zientz E, Feldhaar H, Stoll S, Gross R (2005). Insights into the microbial world associated with ants. Arch Microbiol.

[19] Russell JA, Goldman-Huertas B, Moreau CS, Baldo L, Stahlhut JK, Werren JH (2009). Specialization and geographic isolation among *Wolbachia* symbionts from ants and lycaenid butterflies. Evolution (N Y).

[20] Russell JA, Moreau CS, Goldman-Huertas B, Fujiwara M, Lohman DJ, Pierce NE (2009). Bacterial gut symbionts are tightly linked with the evolution of herbivory in ants. Proc Natl Acad Sci U S A.

[21] Ishikawa H (1989). Biochemical and Molecular Aspects of Endosymbiosis in Insects. Int Rev Cytol.

[22] Sameshima S, Hasegawa E, Kitade O, Minaka N, Matsumoto T (1999). Phylogenetic comparison of endosymbionts with their host ants based on molecular evidence. Zool Sci.

[23] Wernegreen JJ, Kauppinen SN, Brady SG, Ward PS, Douglas A, Baumann P (2009). One nutritional symbiosis begat another: Phylogenetic evidence that the ant tribe Camponotini acquired *Blochmannia* by tending sap-feeding insects. BMC Evol Biol.

[24] Gil R, Silva FJ, Zientz E, Delmotte F, González-Candelas F, Latorre A (2003). The genome sequence of *Blochmannia floridanus*: comparative analysis of reduced genomes. Proc Natl Acad Sci U S A.

[25] Degnan PH, Lazarus AB, Wernegreen JJ (2005). Genome sequence of *Blochmannia pennsylvanicus* indicates parallel evolutionary trends among bacterial mutualists of insects. Genome Res.

[26] Williams LE, Wernegreen JJ (2010). Unprecedented loss of ammonia assimilation capability in a urease-encoding bacterial mutualist. BMC Genomics.

[27] Russell JA, Funaro CF, Giraldo YM, Goldman-Huertas B, Suh D, Kronauer DJC, et al. A veritable menagerie of heritable bacteria from ants, butterflies, and beyond: broad molecular surveys and a systematic review. PLoS One. 2012;7:e51027. 10.1371/journal.pone.0051027 PMC3527441 23284655

[28] Kautz S, Rubin BER, Moreau CS (2013). Bacterial infections across the ants: frequency and prevalence of *Wolbachia, Spiroplasma*, and *Asaia*. Psyche A J Entomol.

[29] Moreau CS. A practical guide to DNA extraction, PCR, and gene-based DNA sequencing in insects. Halteres. 2014;5:32–42.

[30] Caporaso JG, Lauber CL, Walters WA, Berg-Lyons D, Huntley J, Fierer N (2012). Ultra-high-throughput microbial community analysis on the Illumina HiSeq and MiSeq platforms. ISME J.

[31] Ramalho MO, Bueno OC, Moreau CS. Data from this study: Determining the drivers of bacterial community composition in spiny ants (Hymenoptera: Formicidae: Polyrhachis). [Internet]. Figshare Repos. 2016. Available from: https://figshare.com/s/290531bea3dee984444e.

[32] Ramalho M, Bueno O, Moreau C. Camponotini Microbiome. NCBI Seq. Read Arch. under Access. number SRR5136256 study SRP095836. 2016.

[33] Caporaso JG, Kuczynski J, Stombaugh J, Bittinger K, Bushman FD, Costello EK (2010). QIIME allows analysis of high-throughput community sequencing data. Nat Methods.

[34] Quast C, Pruesse E, Yilmaz P, Gerken J, Schweer T, Yarza P (2013). The SILVA ribosomal RNA gene database project: improved data processing and web-based tools. Nucleic Acids Res.

[35] Yilmaz P, Parfrey LW, Yarza P, Gerken J, Pruesse E, Quast C (2014). The SILVA and “All-species Living Tree Project (LTP)” taxonomic frameworks. Nucleic Acids Res.

[36] Edgar RC (2010). Search and clustering orders of magnitude faster than BLAST. Bioinformatics.

[37] Caporaso J, Bittinger K, Bushman F (2010). PyNAST: a flexible tool for aligning sequences to a template alignment.

[38] McArdle BH, Anderson MJ (2001). Fitting multivariate models to community data: a comment on distance-based redundancy analysis. Ecology.

[39] Lozupone C, Knight R (2005). UniFrac: a new phylogenetic method for comparing microbial communities. Appl Environ Microbiol.

[40] McCune B, Grace JB (2002). Analysis of Ecological Communities (MjM Software Design).

[41] Clarke KR (1993). Non-parametric multivariate analyses of changes in community structure. Austral Ecol.

[42] Hammer Ø, Harper D. A. T. & Ryan PD. Paleontological statistics software: package for education and data analysis. Palaeontol Electron. 2001;4:9.

[43] Ramette A. Multivariate analyses in microbial ecology. FEMS Microbiol Ecol. 2007;62:142–60. 10.1111/j.1574-6941.2007.00375.x PMC2121141 17892477

[44] Shannon P, Markiel A, Ozier O, Baliga NS, Wang JT, Ramage D (2003). Cytoscape: a software environment for integrated models of biomolecular interaction networks. Genome Res.

[45] Ley RE, Hamady M, Lozupone C, Turnbaugh PJ, Ramey RR, Bircher JS (2008). Evolution of mammals and their gut microbes. Science.

[46] Oksanen J, Kindt R, Legendre P, O’Hara B. The vegan package. Community Ecol. 2007;10:631–37.

[47] R Development Core Team (2015) R: A language and environment for statistical computing. Available from http://www.R-project.org/. 2016.

[48] Ramalho MO, Martins C, Silva LM, Martins VG, Bueno OC. Intracellular symbiotic bacteria of *Camponotus textor*, Forel (Hymenoptera, Formicidae). Curr Microbiol. 2017;1–9. doi:10.1007/s00284-017-1201-6. 10.1007/s00284-017-1201-6 28261755

[49] Bivand R, Pebesma E, Gomez-Rubio V. Spatial Data Import and Export. Spat Data Anal with R. 2013;83–125.

[50] Kautz S, Rubin BER, Russell JA, Moreau CS (2013). Surveying the microbiome of ants: comparing 454 pyrosequencing with traditional methods to uncover bacterial diversity. Appl Environ Microbiol.

[51] Hu Y, Łukasik P, Moreau CS, Russell JA (2014). Correlates of gut community composition across an ant species (*Cephalotes varians*) elucidate causes and consequences of symbiotic variability. Mol Ecol.

[52] Sanders JG, Powell S, Kronauer DJC, Vasconcelos HL, Frederickson ME, Pierce NE. Stability and phylogenetic correlation in gut microbiota: Lessons from ants and apes. Mol Ecol. 2014;23:1268–83. 10.1111/mec.12611 24304129

[53] Linnenbrink M, Wang J, Hardouin EA, Künzel S, Metzler D, Baines JF (2013). The role of biogeography in shaping diversity of the intestinal microbiota in house mice. Mol Ecol.

[54] Winston ME, Hampton-Marcell J, Zarraonaindia I, Owens SM, Moreau CS, Gilbert JA, et al. Understanding cultivar-specificity and soil determinants of the *Cannabis* microbiome. PLoS One. 2014;9:e99641. 10.1371/journal.pone.0099641 PMC4059704 24932479

[55] Jones RRT, Sanchez LG, Fierer N, Dale C, Moran N, Lamelas A (2013). A cross-taxon analysis of insect-associated bacterial diversity. Gilbert JA, editor. PLoS One.

[56] Theis KR, Schmidt TM, Holekamp KE, Rosenberg E, Sharon G, Atad I (2012). Evidence for a bacterial mechanism for group-specific social odors among hyenas. Sci Rep.

[57] Colman DR, Toolson EC, Takacs-Vesbach CD (2012). Do diet and taxonomy influence insect gut bacterial communities?. Mol Ecol.

[58] Santo Domingo JW, Kaufman MG, Klug MJ, Holben WE, Harris D, Tiedje JM (1998). Influence of diet on the structure and function of the bacterial hindgut community of crickets. Mol Ecol.

[59] Schmitt-Wagner D, Friedrich MW, Wagner B, Brune A (2003). Axial dynamics, stability, and interspecies similarity of bacterial community structure in the highly compartmentalized gut of soil-feeding termites (*Cubitermes* spp.). Appl Environ Microbiol.

[60] Hongoh Y, Deevong P, Inoue T, Moriya S, Trakulnaleamsai S, Ohkuma M (2005). Intra- and interspecific comparisons of bacterial diversity and community structure support coevolution of gut microbiota and termite host. Appl Environ Microbiol.

[61] Mohr KI, Tebbe CC (2006). Diversity and phylotype consistency of bacteria in the guts of three bee species (Apoidea) at an oilseed rape field. Environ Microbiol.

[62] Behar A, Yuval B, Jurkevitch E (2008). Community structure of the mediterranean fruit fly microbiota: seasonal and spatial sources of variation. Isr J Ecol Evol.

[63] Anderson KE, Russell JA, Moreau CS, Kautz S, Sullam KE, Hu Y (2012). Highly similar microbial communities are shared among related and trophically similar ant species. Mol Ecol.

[64] Dillon RJ, Dillon VM (2004). The gut bacteria of insects: Nonpathogenic Interactions. Annu Rev Entomol.

[65] Engel P, Moran NA. The gut microbiota of insects – diversity in structure and function. FEMS Microbiol Rev. 2013;37 10.1111/1574-6976.12025 23692388

[66] Jones RT, Bressan A, Greenwell AM, Fierer N (2011). Bacterial communities of two parthenogenetic aphid species cocolonizing two host plants across the Hawaiian Islands. Appl Environ Microbiol.

[67] Andert J, Marten A, Brandl R, Brune A. Inter- and intraspecific comparison of the bacterial assemblages in the hindgut of humivorous scarab beetle larvae (Pachnoda spp.). FEMS Microbiol Ecol. 2010;74. 10.1111/j.1574-6941.2010.00950.x 20738398

[68] Moran NA, Hansen AK, Powell JE, Sabree ZL, Cox-Foster D, Conlan S (2012). Distinctive gut microbiota of honey bees assessed using deep sampling from individual worker bees. Smagghe G, editor. PLoS One.

[69] Jones RT, Bernhardt SA, Martin AP, Gage KL. Interactions among symbionts of *Oropsylla* spp. (Siphonoptera: Ceratophyllidae). J Med Entomol. 2012;49:492–96. 10.1603/me11244 22679855

[70] Munson MA, Baumann P, Clark MA, Baumann L, Moran NA, Voegtlin DJ (1991). Evidence for the establishment of aphid-eubacterium endosymbiosis in an ancestor of four aphid families. J Bacteriol.

[71] Baumann P. Biology of bacteriocyte-associated endosymbionts of plant sap-sucking insects. Annu Rev. Microbiol. 2005;59:155–89. 10.1146/annurev.micro.59.030804.121041 16153167

[72] Wu D, Daugherty SC, Van Aken SE, Pai GH, Watkins KL, Khouri H (2006). Metabolic complementarity and genomics of the dual bacterial symbiosis of sharpshooters. Parkhill J, editor. PLoS Biol.

[73] Feldhaar H, Straka J, Krischke M, Berthold K, Stoll S, Mueller MJ (2007). Nutritional upgrading for omnivorous carpenter ants by the endosymbiont *Blochmannia*. BMC Biol.

[74] Wolschin F, Hölldobler B, Gross R, Zientz E (2004). Replication of the endosymbiotic bacterium *Blochmannia floridanus* is correlated with the developmental and reproductive stages of its ant host. Appl Environ Microbiol.

[75] De Souza DJ, Bézier A, Depoix D, Drezen J-M, Lenoir A (2009). *Blochmannia* endosymbionts improve colony growth and immune defence in the ant *Camponotus fellah*. BMC Microbiol.

[76] Fan Y, Thompson JW, Dubois LG, Moseley MA, Wernegreen JJ (2013). Proteomic analysis of an unculturable bacterial endosymbiont (*Blochmannia*) reveals high abundance of chaperonins and biosynthetic enzymes. J Proteome Res.

[77] Degnan PH, Lazarus AB, Brock CD, Wernegreen JJ (2004). Host-symbiont stability and fast evolutionary rates in an ant-bacterium association: cospeciation of *Camponotus* species and their endosymbionts, *Candidatus Blochmannia*. Syst Biol.

[78] Brown BP, Wernegreen JJ. Deep divergence and rapid evolutionary rates in gut-associated Acetobacteraceae of ants. BMC Microbiol. 2016;16:140. 10.1186/s12866-016-0721-8 PMC4939635 27400652

[79] Rideout JR, He Y, Navas-Molina JA, Walters WA, Ursell LK, Gibbons SM (2014). Subsampled open-reference clustering creates consistent, comprehensive OTU definitions and scales to billions of sequences. PeerJ.

[80] Kellner K, Ishak HD, Linksvayer TA, Mueller UG. Bacterial community composition and diversity in an ancestral ant fungus symbiosis. FEMS Microbiol Ecol. 2015;91:fiv073. 10.1093/femsec/fiv073 26113689

[81] Hammes WP, Vogel RF. The genus *Lactobacillus*. Genera Lact. Acid Bact. Boston, MA: Springer US; 1995.

[82] Long YH, Xie L, Liu N, Yan X, Li MH, Fan MZ (2010). Comparison of gut-associated and nest-associated microbial communities of a fungus-growing termite (*Odontotermes yunnanensis*). Insect Sci.

[83] Mathew GM, Ju Y-M, Lai C-Y, Mathew DC, Huang CC. Microbial community analysis in the termite gut and fungus comb of *Odontotermes formosanus*: the implication of *Bacillus* as mutualists. FEMS Microbiol Ecol. 2012;79:504–17. 10.1111/j.1574-6941.2011.01232.x 22092951

[84] Hölldobler B, Engel-Siegel H (1984). On the metapleural gland of ants. Psyche A J Entomol.

[85] Yek SH, Mueller UG. The metapleural gland of ants. Biol Rev. 2011;86:774–91. 10.1111/j.1469-185X.2010.00170.x 21504532

[86] Johnson RN, Agapow P-MM, Crozier RH (2003). A tree island approach to inferring phylogeny in the ant subfamily Formicinae, with especial reference to the evolution of weaving. Mol Phylogenet Evol.

[87] Graystock P, Hughes WOH (2011). Disease resistance in a weaver ant, *Polyrhachis dives,* and the role of antibiotic-producing glands. Behav Ecol Sociobiol.

[88] Meirelles LA, McFrederick QS, Rodrigues A, Mantovani JD, de Melo RC, Ferreira H (2016). Bacterial microbiomes from vertically transmitted fungal inocula of the leaf-cutting ant *Atta texana*. Environ Microbiol Rep.

